# Identification of Key Genes and Pathways Involved in Circulating Tumor Cells in Colorectal Cancer

**DOI:** 10.1155/2022/9943571

**Published:** 2022-01-27

**Authors:** Ruijun Pan, Chaoran Yu, Yanfei Shao, Hiju Hong, Jing Sun, Zhou Zhang, Peiyong Li, Minhua Zheng

**Affiliations:** ^1^Department of General Surgery, Ruijin Hospital, Shanghai Jiao Tong University School of Medicine, Shanghai Minimally Invasive Surgery Center, Shanghai 200025, China; ^2^Department of Nuclear Medicine, Ruijin Hospital, Shanghai Jiao Tong University School of Medicine, Shanghai 200025, China; ^3^Department of General Surgery, Shidong Hospital Yangpu District, Shanghai 200025, China

## Abstract

**Background:**

Characterization of the features associated with circulating tumor cells (CTCs) is one of major interests for predicting clinical outcome of colorectal cancer (CRC) patients. However, the molecular features of CTCs remain largely unclear.

**Methods:**

For identification of key genes and pathways, GSE31023, contained CTCs from six metastatic CRC patients and three controls, was retrieved for differentially expressed gene (DEG) analysis. Protein-protein interaction networks of DEGs were constructed. Hub genes from the network were prognostic analyzed, as well as the association with tumor-infiltrating immune cells.

**Results:**

1353 DEGs were identified between the CTC and control groups, with 403 genes upregulated and 950 downregulated. 32 pathways were significantly enriched in KEGG, with ribosome pathway as top. The top 10 hub genes were included, including eukaryotic translation elongation factor 2 (EEF2), ribosomal protein S2 (RPS2), ribosomal protein S5 (RPS5), ribosomal protein L3 (RPL3), ribosomal protein S3 (RPS3), ribosomal protein S14 (RPS14), ribosomal protein SA (RPSA), eukaryotic translation elongation factor 1 alpha 1 (EEF1A1), ribosomal protein S15a (RPS15A), and ribosomal protein L4 (RPL4). The correlation between CD4^+^ T cells and RPS14 (correlation = −0.5) was the highest in colon cancer while CD8^+^ T and RPS2 (correlation = −0.53) was the highest in rectal cancer.

**Conclusion:**

This study identified potential role of ribosome pathway in CTC, providing further insightful therapeutic targets and biomarkers for CRC.

## 1. Introduction

Colorectal cancer (CRC) is one of the major digestive malignancies in the world. During the tumor progression, hematogenous tumor cell disseminates and initiates the metastatic cascade of CRC. Circulating tumor cells (CTCs) exist in the peripheral blood of patients with various solid tumors including colorectal cancer and may lead to tumor metastasis [1]. With the development of liquid biopsy, CTCs have been proven to play an important role in detecting early development of metastasis and monitoring the curative effect of adjuvant therapy [2]. Therefore, molecular characterization of CTCs has been one of major interests for predicting clinical outcome of patients [3].

Due to the low concentration of CTCs in blood, their detection needs highly sensitive and specific methods, including separation (concentration) and identification (detection). At present, CTCs and peripheral hematopoietic cells are generally distinguished according to their biological characteristics (expression and activity of cell surface proteins) and physical characteristics (size, density, charge, and deformability). Compared to the diameter of the blood cells (8 *μ*m), tumor cells are larger and less likely to deform. Based on these characteristics, many membrane filtration devices appeared for CTC enrichment, including microelectromechanical system- (MEMS-) opticbased microfilter, isolation by size of epithelial tumor cells (ISET), CellSieve™, ScreenCell®, and CellOptics® [4]. However, the morphological method to distinguish tumor cells from blood cells lacks certain specificity, and some smaller CTCs may be lost. Thus, immunocytochemistry and nucleic acid technology, highly sensitive and specific methods, have been commonly used to identify CTCs by detecting surface biomarkers with distinguished expression. Epithelial cell adhesion molecule (EpCAM), the most used antigen in CTCs, has been proven to be one of the key molecules associated with Wnt signaling pathway and cellular adhesion [5, 6]. During the initiation of spread, profile-changed tumor cells were increased in bloodstream with improved risk to form secondary tumor. At the origin of metastasis, EpCAM expression was absent in some cells due to epithelial-to-mesenchymal transition (EMT) process, while emerged again with activated mesenchymal-to-epithelial transition (MET) when metastatic lesions have been formed [7–9]. CTCs can undergo EMT and MET processes with a wide spectrum of CTC phenotypes in the bloodstream. Thus, the isolation of CTC-based solely measurement of EpCAM expression remains challenging to the isolation of CTCs. More markers are needed for higher yield of CTCs [10, 11].

Epidermal growth factor receptor (EGFR), a transmembrane receptor involved in multiple biological processes, has also been regarded as a specific marker of CTCs. Analysis of EGFR status in collected CTCs prior to treatment could potentially be benefit for the patients to select an appropriated targeted therapy. It has been reported that examining mutation of CTC levels in non-small-cell lung cancer (NSCLC) may be helpful in detecting heterogenic mutations in EGFR [12]. In fact, the usage of EGFR in CTCs remains limited due to the limited benefits of targeted therapy.

Collectively, single biomarker could not delineate the whole picture of CTCs with the molecular features yet to be fully characterized. Given the increasing clinical practice and prognostic values of CTCs, this study employed GSE31023 [13], containing six CTC samples from metastatic CRC patients with three normal controls, to identify potential key genes and pathways associated with CTCs of CRC.

## 2. Materials and Methods

### 2.1. Methods

#### 2.1.1. Gene Expression Profile GSE31023 for Analysis

GSE31023 was the gene expression profiling by array, and all corresponding data was downloaded from the Gene Expression Omnibus (GEO) database (http://www.ncbi.nlm.nih.gov/geo/) [13]. This profile contained CTCs from six metastatic CRC patients and three healthy donors as control. And the related CTCs were isolated from 7.5 mL of peripheral blood by immunomagnetic separation using anti-EpCAM-coated magnetic beads (). Briefly, RNA in each sample was extracted and amplified using a whole transcriptome amplification system [13]. GPL13497 (Agilent-026652 Whole Human Genome Microarray 4∗44K v2) was the platform for GSE31023.

#### 2.1.2. Functional Annotation of Differentially Expressed Genes (DEGs)

The DEGs between the CTCs and normal cells were identified using the web tool, GEO2R, with predefined cutoff value *p* value < 0.05 and ∣log fold change (logFC) | >1 [14]. The gene ontologies (GOs), as well as the Kyoto Encyclopedia of Genes and Genomes (KEGG) analysis, were employed for selected DEGs using the Database for Annotation, Visualization, and Integrated Discovery platform (DAVID, http://david.abcc.ncifcrf.gov/) [15–18]. Top 10 terms in each category, including biological process (BP), cellular component (CC), and molecular function (MF), were displayed if more than 10 terms were defined as significant (*p* value < 0.05).

#### 2.1.3. Construction of Protein-Protein Interaction (PPI) Networks

PPI networks of DEGs were performed using the Search Tool for the Retrieval of Interacting Genes/Proteins (STRING, http://www.string-db.org/) and visualized by the Cytoscape software (version 3.6.0) [19, 20]. Moreover, the Molecular Complex Detection (MCODE) program was used for subcluster identification of the PPI [21]. BiNGO program was used for the GO presentation in the network analysis [22]. Hub genes were defined as the ten genes with highest degree determined by the PPI network.

#### 2.1.4. Expression of Hub Genes in The Cancer Genome Atlas (TCGA)

The mRNA expression boxplot of hub genes of TCGA (colon cancer, COAD and rectal cancer, READ) was retrieved from the gene expression profiling interactive analysis platform (GEPIA, http://gepia.cancer-pku.cn) [23].

### 2.2. Correlation of Tumor-Infiltrating Immune Cells (TIICs) and Hub Genes

Tumor Immune Estimation Response (TIMER, https://cistrome.shinyapps.io/timer/) is a novel platform for analyzing the expression abundance of the immune infiltration cells (CD8^+^ T cells, CD4^+^ T cells, dendritic cells, macrophages, neutrophils, and B cells) in malignant tumors, which was set up for online comparison based on references in TCGA [24]. Thus, the correlation of hub genes and all immune cells related in tumor was explored via TIMER. The correlation value was corrected by tumor purity [24].

### 2.3. Prognostic Values of Hub Gene Signature Defined Risk Groups

The prognostic values of hub gene signature defined risk groups in both overall survival (OS) and disease-free survival (DFS) were explored via the SurvExpress platform (http://bioinformatica.mty.itesm.mx:8080/Biomatec/SurvivaX.jsp) [25]. High- and low-risk groups were determined based on the risk score algorithm [25].

## 3. Results

### 3.1. Identification and Functional Enrichment Analysis of DEGs

**Figure 1:**
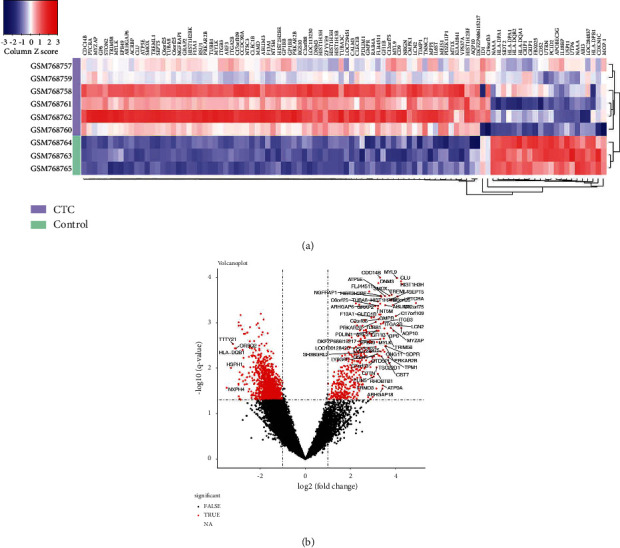
Identification of differentially expressed genes (DEGs) between circulating tumor cells (CTCs) and normal control groups. (a) The identified DEGs displayed in heat map. (b) The identified DEGs displayed by volcano plot.

**Figure 2:**
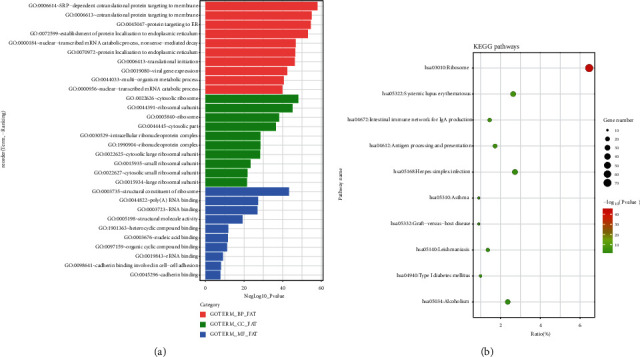
Enrichment analysis of DEGs. (a) Gene ontologies and DEGs; (b) Kyoto Encyclopedia of Genes and Genomes enrichment of DEGs.

A total of 1353 DEGs were identified between the CTCs and control groups, with 403 genes upregulated and 950 downregulated (Figures 1(a) and 1(b)). A total of 547 BP terms were significantly enriched. The most enriched three terms in BP were SRP-dependent cotranslational protein targeting to membrane, cotranslational protein targeting to membrane, and protein targeting to ER. A total of 142 terms were significantly enriched in CC. The most enriched three terms in CC were cytosolic ribosome, ribosomal subunit, and ribosome. A total of 100 terms were significantly enriched in MF. The most enriched three terms in MF were structural constituent of ribosome, poly (A) RNA binding, and RNA binding (Figure 2(a)). Noteworthy, a total of 32 pathways were significantly enriched in KEGG. The top three were ribosome (false discovery rate, FDR = 6.41*E* − 42), systemic lupus erythematosus (FDR =2.39E-04), and intestinal immune network for IgA production (FDR = 6.28*E* − 04) (Figure 2(b)).

### 3.2. PPI Network Establishment of DEGs

**Figure 3:**
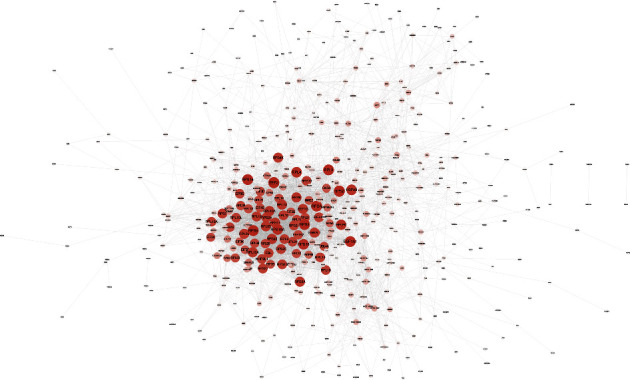
The protein-protein interaction (PPI) networks and the network analysis of enrichment results of DEGs. Nodes indicated each DEG. Lines represented in-between interactions. The size of each node and density of color were in proportion to the degrees.

**Figure 4:**
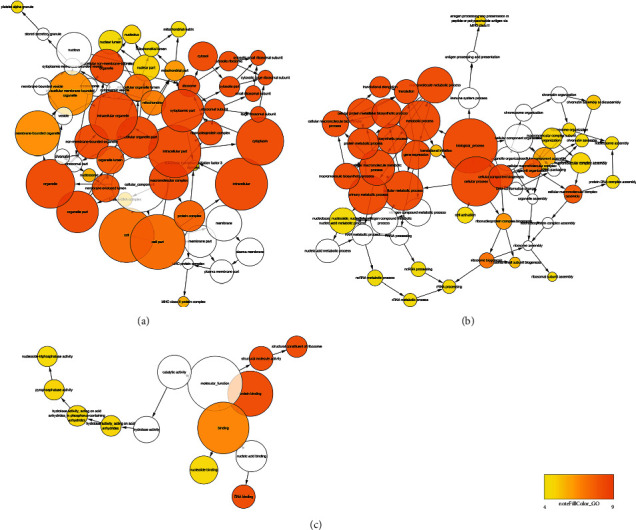
The network analysis of enrichment results of DEGs. (a) The enrichment of cellular components. (b) The enrichment of biological processes and the density of color were in proportion to the significance. (c) The network analysis of enrichment results of molecular functions by DEGs. The density of color was in proportion to the significance.

Next, we explored the PPI network of all DEGs. In fact, a total of 496 nodes and 4283 edges were identified within the PPI network (Figure 3). Meanwhile, the functional enrichment network was also displayed (Figures 4(a)–4(c)). The top 10 hub genes include deukaryotic translation elongation factor 2 (EEF2), ribosomal protein S2 (RPS2), ribosomal protein S5 (RPS5), ribosomal protein L3 (RPL3), ribosomal protein S3 (RPS3), ribosomal protein S14 (RPS14), ribosomal protein SA (RPSA), eukaryotic translation elongation factor 1 alpha 1 (EEF1A1), ribosomal protein S15a (RPS15A), and ribosomal protein L4 (RPL4) (Table 1). The top three scored modules were determined by MCODE and further functionally enriched, which also highlighted the role of ribosome (Table 2). Noteworthy, all the hub genes were found downregulated in CTCs.

### 3.3. Expression of Hub Genes

**Figure 5:**
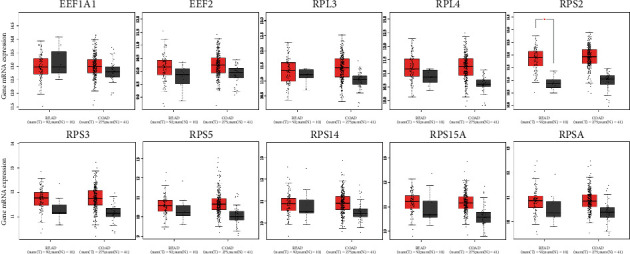
The mRNA expression of hub genes between tumor and normal using the GEPIA platform. Boxplot was used for mRNA expression between tumor (red) and normal (grey). READ: rectal adenocarcinoma; COAD: colon adenocarcinoma.

**Figure 6:**
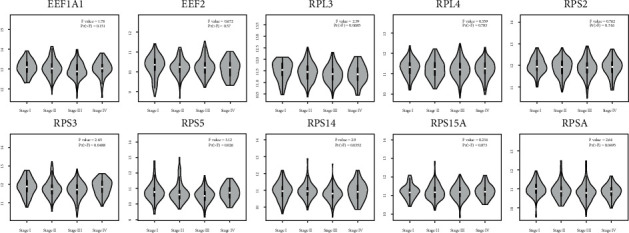
The stage-specific mRNA expression of hub genes by violin plot using the GEPIA platform. READ: rectal adenocarcinoma; COAD: colon adenocarcinoma; tumor: red; normal: grey.

Of all the expression comparison between tumor and normal, only RPS2 was upregulated in tumor compared to normal in READ (Figure 5). RPS3, RPS5, RPS14, and RPSA were found significantly stage-specific expressed (Figure 6).

### 3.4. The Correlation between Hub Genes and TIICs

**Figure 7:**
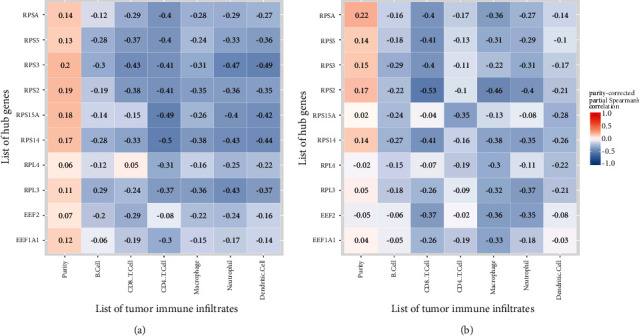
The correlation between hub genes and tumor-infiltrating immune cells (TIICs). (a) The correlation displayed in colon cancer of The Cancer Genome Atlas (TCGA); (b) the correlation displayed in rectal cancer of TCGA. Blue indicated negative purity-corrected partial Spearman's correlation while red indicated positive correlation.

Furthermore, the correlation between hub genes and TIICs was analyzed via the TIMER platform. In colon cancer, the highest correlation was found between CD4^+^ T cells and RPS14 (correlation = −0.5) and CD4^+^ T and RPS15A (correlation = −0.49), as well as dendritic cells and RPS3 (correlation = −0.49). In rectal cancer, the highest correlation was found between CD8^+^ T and RPS2 (correlation = −0.53) and macrophage and RPS2 (correlation = −0.46) (Figure 7).

### 3.5. Prognostic Values of Hub Gene Signature

**Figure 8:**
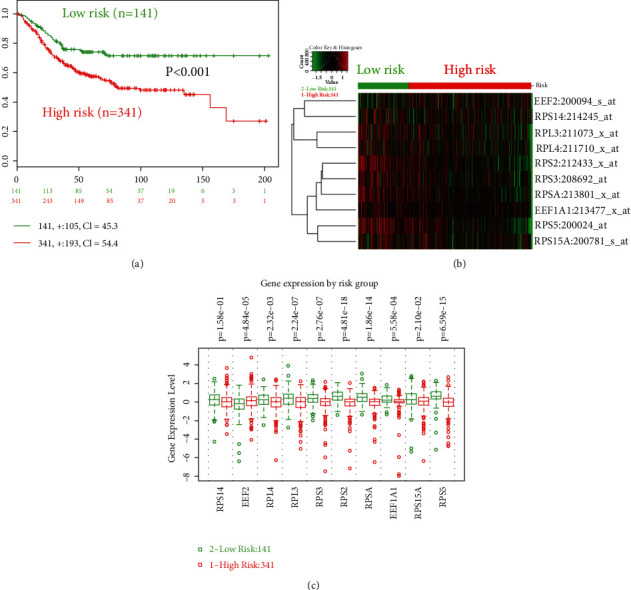
The prognostic values (overall survival, OS) of hub gene signature in colorectal cancer via the SurvExpress platform. (a) The KM plot of high-/low-risk groups divided by prognostic index; (b) the mRNA expression of high-/low-risk OS groups in heat map; (c) the mRNA expression comparison between high- and low-risk OS groups. High risk: red; low risk: green.

**Figure 9:**
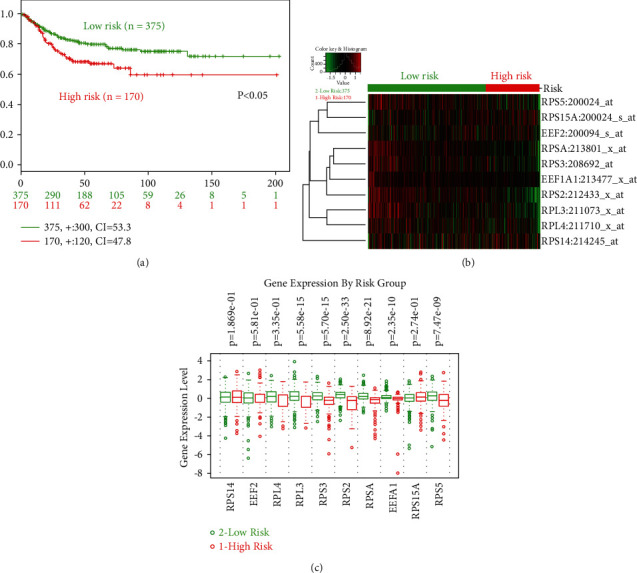
The prognostic values (disease-free survival, DFS) of hub gene signature in colorectal cancer via the SurvExpress platform. (a) The KM plot of high-/low-risk groups divided by prognostic index; (b) the mRNA expression of high-/low-risk DFS groups in heat map; (c) the mRNA expression comparison between high- and low-risk DFS groups. High risk: red; low risk: green.

Given increasing focus has been found in the prognostic roles of gene signature, this study further explored the prognostic values of hub gene signature via the SurvExpress platform. In OS analysis, significant prognostic roles were found between high-risk and low-risk groups (hazard ratio = 1.99, 95% confidence interval: 1.38-2.87, and *p* = 0.0002) (Figure 8(a)). Meanwhile, the expression comparison was also illustrated between two groups (Figures 8(b) and 8(c)). In DFS analysis, significant prognostic roles were also found between high-risk and low-risk groups (hazard ratio = 1.71, 95% confidence interval: 1.2-2.46, and *p* = 0.003) with expression comparison (Figure 9).

## 4. Discussion

Commonly, standard patterns for the detection of CTCs in CRC are closely associated with genomic features. In fact, the intrinsic genomic features of metastatic lesions may not be identical to those of primary lesions [26]. During the metastatic progression, tumor cells show reduced adhesion markers and gradually detach from the primary lesion and flow into the circulation system. However, not all of the CTCs could be successfully habited at distant organs. Only a small proportion of tumor cells survives the intrinsic immunological eradication and undergoes profile-change at the secondary lesion. Meanwhile, normal epithelial cells also could join the circulated traveling, guided by inflammation-triggered cytokines [27]. Thus, molecular characterization of CTCs is needed. However, the reculture of isolated CTCs remains technically difficult. Zhang et al. reported that a population of CTCs from 3 patients with breast cancer could be successfully used to form adherent cell line, with limited survival period and proliferation status [28]. Guan et al. have analyzed 7 GEO datasets (GSE99394, GSE31023, GSE82198, GSE65505, GSE67982, GSE76250, and GSE50746) and found that CTCs mainly change epithelial-mesenchymal transition (EMT), cell adhesion, and apoptosis [29]. Based on the study, we further indicated the key genes and pathways mainly involved in CTCs in CRC and revealed more promising biomarkers in CRC prognosis and immunotherapy.

Noteworthy, ribosome pathway was highlighted in this study given the enrichment analysis of DEGs between CTCs and control. Interestingly, most of the hub genes were closely associated with ribosome pathway and all downregulated in CTCs compared to control. Consistently, expression profiling of breast cancer also highlighted the ribosome-related pathways and terms in genes downregulated in CTCs compared to control [30]. In fact, reduced levels of immune signals and apoptotic pathways were also enriched in CTCs of breast cancer [30]. Moreover, mammalian target of rapamycin pathway, constitutively activated by upstream AKT and PI3K pathways, was one of the key targets for persistent/recurrent epithelial ovarian cancer and closely associated with ribosome protein and eukaryotic translation initiation factor [31]. This study highlighted potential role of ribosome in CTCs of CRC, and the analysis of hub genes has opened up a new question as the therapeutic value of ribosome in CTCs.

For 10 hub genes, remarkable correlations with TIICs and prognostic values had been recognized in this study. However, solid validation remained in another independent CTC cohort, instead of conventional tissue-based genome results. Furthermore, only RPS2 was upregulated in tumor compared to normal in rectal cancer of TCGA, which may due to the different molecular expression characteristics between CTCs and solid tumor cells. Therefore, it is reasonable to further validate the results in an independent CTC cohort study.

Our study had the following strengths. First, we further identified the differentially expressed genes and pathways involved in CTCs in CRC. Second, several external datasets were used to verify that these hub genes can be related to the prognosis and immunotherapy of CRC patients. Besides, the study also has some limitations. First, the databases retrieving data from studies were conducted in different ways. Second, the direct relationship between these hub genes in CTCs and clinical characteristics has not been further verified.

## 5. Conclusion

This study identified potential role of ribosome pathway in CTC, providing further insightful therapeutic targets for CRC. Moreover, the association between hub genes and CTCs may provide new perspectives for the exploit of new markers.

## Figures and Tables

**Table 1:** Hub genes identified by the protein-protein interaction (PPI) networks.

Gene symbol	Log FC	Expression^∗^	Gene name
EEF2	-1.86	Down	Eukaryotic translation elongation factor 2
RPS2	-1.76	Down	Ribosomal protein S2
RPS5	-2.00	Down	Ribosomal protein S5
RPL3	-1.92	Down	Ribosomal protein L3
RPS3	-1.58	Down	Ribosomal protein S3
RPS14	-1.53	Down	Ribosomal protein S14
RPSA	-2.35	Down	Ribosomal protein SA
EEF1A1	-1.88	Down	Eukaryotic translation elongation factor 1 alpha 1
RPS15A	-1.46	Down	Ribosomal protein S15a
RPL4	-1.55	Down	Ribosomal protein L4

^∗^Gene expression in CTC compared to normal control.

**Table 2:** Top scored three Molecular Complex Detection (MCODE) clusters and significantly enriched pathways. FDR: false discovery rate.

MCODE	Genes	Interactions	Gene symbols	Enriched pathways (FDR < 0.05)
1	61	1791	RPS27, RPS23, RPS14, RPL10L, RPL10, EIF3D, EIF3B, RPL24, RPS21, RPSA, NHP2L1, FBL, RPL7A, RPS16, RPS10, RPL19, RPL13, RPL4, RPS7, RPS3, RPL38, RPS26, RPL6, RPS4X, RPS3A, RPL18, RPL29, RPL14, RPS5, RPL35, RPS25, RPL3, RPL10A, RPS24, RPS15A, RPL12, RPL26, RPS19, RPL32, RPL18A, RPS11, RPL15, RPL36, RPL22, RPLP0, RPS2, RPL21, RPL13A, EIF4B, NSA2, EIF3K, EIF3F, EIF4A1, GNB2L1, EIF3A, EEF1B2, EIF3G, EEF1A1, EEF1G, EEF2, RPS6	hsa03010:ribosome

2	14	45	SPARC, GABBR1, TIMP1, CNR2, GNG7, CCL5, TGFB1, GNG11, GNG8, F13A1, P2RY12, CLU, ITIH4, ACTN1	NA

3	28	86	HLA-DPA1, HLA-DPB1, LSM7, HIST1H2AM, DNM3, CTTN, HIST1H2AI, HIST1H2BF, HLA-DRB1, HLA-DRB5, CIITA, H2AFJ, HIST1H2BK, HIST1H2AA, HIST1H2BB, HIST1H2AE, HIST2H2BE, RAN, HLA-DMA, DAB2, LDLRAP1, HLA-DQA1, SNRPD1, REPS1, HLA-DQB1, STON2, PPIH, RBMX	hsa05322:systemic lupus erythematosus hsa05310:asthma hsa05332:graft-versus-host disease hsa05330:allograft rejection hsa04940:type I diabetes mellitus hsa04612:antigen processing and presentation hsa04672:intestinal immune network for IgA production hsa05320:autoimmune thyroid disease hsa05150:Staphylococcus aureus infection hsa05416:viral myocarditis hsa05321:inflammatory bowel disease (IBD) hsa05145:toxoplasmosis hsa05034:alcoholism hsa05140:leishmaniasis hsa05323:rheumatoid arthritis hsa05164:influenza A hsa05152:tuberculosis hsa04514:cell adhesion molecules (CAMs) hsa04145:phagosome hsa05166:HTLV-I infection hsa05168:herpes simplex infection

## Data Availability

The datasets supporting the conclusion of this article were included within the article.

## Abbreviations

CRC: Colorectal cancer
CTCs: Circulating tumor cells
EMT: Epithelial-to-mesenchymal transition process
MET: Mesenchymal-to-epithelial transition
ISET: Isolation by size of epithelial tumor cells
EpCAM: Epithelial cell adhesion molecule
EGFR: Epidermal growth factor receptor
GEO: Gene Expression Omnibus
GOs: Gene ontologies
KEGG: Kyoto encyclopedia of genes and genomes
DAVID: Database for Annotation, Visualization, and Integrated Discovery platform
BP: Biological process
CC: Cellular component
MF: Molecular function
PPI: Protein-protein interaction
STRING: Search Tool for the Retrieval of Interacting Genes/Proteins
MCODE: Molecular Complex Detection
TCGA: The Cancer Genome Atlas
COAD: Colon cancer
READ: Rectal cancer
GEPIA: Gene expression profiling interactive analysis platform
TIICs: Tumor-infiltrating immune cells
TIMER: Tumor immune estimation response
DFS: Disease-free survival
OS: Overall survival.
